# Succinyl-CoA Synthetase Dysfunction as a Mechanism of Mitochondrial Encephalomyopathy: More than Just an Oxidative Energy Deficit

**DOI:** 10.3390/ijms241310725

**Published:** 2023-06-27

**Authors:** Makayla S. Lancaster, Brett H. Graham

**Affiliations:** Department of Medical & Molecular Genetics, Indiana University School of Medicine, 975 W. Walnut St., Room IB257, Indianapolis, IN 46202, USA; andermas@iu.edu

**Keywords:** succinyl-CoA synthetase, tricarboxylic acid cycle, mitochondria, encephalomyopathy, mitochondrial DNA, protein succinylation

## Abstract

Biallelic pathogenic variants in subunits of succinyl-CoA synthetase (SCS), a tricarboxylic acid (TCA) cycle enzyme, are associated with mitochondrial encephalomyopathy in humans. SCS catalyzes the interconversion of succinyl-CoA to succinate, coupled to substrate-level phosphorylation of either ADP or GDP, within the TCA cycle. SCS-deficient encephalomyopathy typically presents in infancy and early childhood, with many patients succumbing to the disease during childhood. Common symptoms include abnormal brain MRI, basal ganglia lesions and cerebral atrophy, severe hypotonia, dystonia, progressive psychomotor regression, and growth deficits. Although subunits of SCS were first identified as causal genes for progressive metabolic encephalomyopathy in the early 2000s, recent investigations are now beginning to unravel the pathomechanisms underlying this metabolic disorder. This article reviews the current understanding of SCS function within and outside the TCA cycle as it relates to the complex and multifactorial mechanisms underlying SCS-related mitochondrial encephalomyopathy.

## 1. Introduction

With no curative treatments currently available, mitochondrial disorders are one of the most common forms of multi-system disease, affecting about 1 in 4300 people [1]. Mitochondria are double membrane-bound cellular organelles conventionally recognized as a significant hub for intermediary metabolism. In addition to its role in oxidative phosphorylation (OXPHOS), the mitochondria are involved in apoptosis, pyrimidine and heme synthesis, calcium ion homeostasis, and intracellular signaling, among other critical biological pathways [2]. While mitochondrial dysfunction can be caused by perturbations in any of these processes, disorders of the mitochondria are classically recognized by a failure of the mitochondria to produce enough cellular ATP to meet energy demands, usually as a result of primary or secondary respiratory chain (RC) dysfunction. Most commonly, organs with high energy demand, such as the brain, heart, and skeletal muscle, are affected; therefore, mitochondrial disease typically manifests as encephalomyopathy, with a wide range of neurological symptoms, including developmental delay, seizures, strokes, and sensorineural hearing loss [3,4].

Although mitochondrial disorders are one of the most common forms of inherited metabolic and neurological disorders, the investigation into their pathogenesis is complicated by wide clinical and genetic heterogeneity. Mitochondria are complex, with the mitochondrial proteome encoded within the nucleus and a localized multicopy genome (mtDNA) encoding 37 genes essential for mitochondrial function, including 13 genes encoding proteins within the mitochondrial RC and ATP synthetase [5,6]. Therefore, mitochondrial disorders can result from pathogenic variants within the nuclear or mitochondrial genome. However, as most of the mitochondrial proteome is encoded by the nuclear DNA (nDNA), many mitochondrial disorders result from nuclear-encoded genes directly or indirectly involved in OXPHOS. With variants in over 300 nuclear-encoded genes associated with mitochondrial disease [7], variants in nuclear-encoded genes comprise most inherited mitochondrial disorders, have earlier disease onset, and are typically more severe, with most patients dying in early childhood [8].

Succinyl-CoA synthetase (SCS) is a nuclear-encoded protein complex within the mitochondrial tricarboxylic acid (TCA) cycle, with biallelic pathogenic variants associated with mitochondrial disease in human patients. SCS catalyzes the reversible conversion of succinyl-CoA to succinate in the only step of the TCA cycle coupled with substrate-level phosphorylation of either ADP or GDP. In eukaryotes, SCS is a heterodimeric complex with a single catalytic alpha subunit, SUCLG1, and two isoforms of the beta subunit, ATP-generating SUCLA2 and GTP-forming SUCLG2. Pathogenic variants in both *SUCLG1* and *SUCLA2* have been identified as causal variants in early-onset mitochondrial encephalomyopathy in humans [9]. Although human variants in SCS were first reported in 2005 [10], investigations into the mechanisms linking this primary TCA cycle dysfunction to mitochondrial disease are incomplete. This review assesses the current and recent literature surrounding SCS and SCS deficiency as an etiology of mitochondrial encephalomyopathy, including the structure and function of SCS within the TCA cycle, the neurological clinical spectrum of SCS deficiency, and the current status of research efforts into pathogenic mechanisms of SCS-deficient mitochondrial disease. With a better understanding of the complex and multidimensional pathogenesis of SCS deficiency, investigators will be better suited to continue research into potential therapeutic options for SCS deficiency and mitochondrial encephalomyopathies.

## 2. The Canonical Structure and Function of Succinyl-CoA Synthetase

### 2.1. Mechanistic Insights from Prokaryotic Research

**Figure 1:**
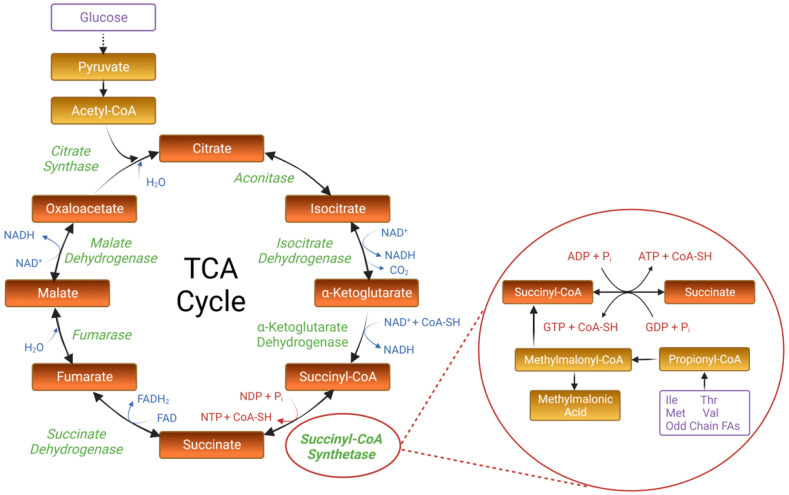
The Role of Succinyl-CoA Synthetase (SCS) Within the Tricarboxylic (TCA) Cycle. The full TCA cycle presented was generated in *BioRender*. Metabolic intermediates are boxed in orange, and catalytic enzymes are provided in green. SCS, circled in red, catalyzes the fifth step of the TCA cycle, the interconversion of succinyl-CoA to succinate coupled with substrate-level phosphorylation. A close-up of metabolic pathways that feed into the TCA cycle via succinyl-CoA is provided, including amino acid pathways and fatty acid catabolism.

**Figure 2:**
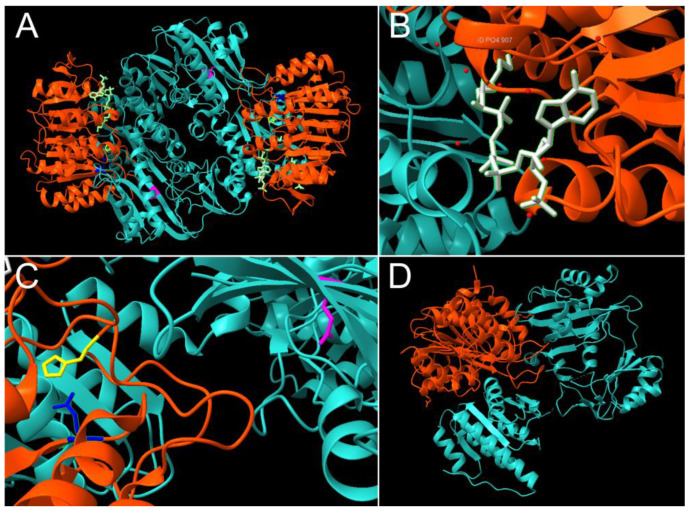
SCS Structure in *E. coli* and Humans. (**A**) The heterotetrameric structure of SCS in *E. coli* with alpha subunits in orange and beta subunits in teal (PDB ID: 1CQJ) [15]. Close-up images of the succinyl-CoA (white) binding site (**B**) and catalytic residues (**C**) within the nucleotide-binding site are provided. His246α is shown in yellow, Glu208α in blue and Glu197β in purple. (**D**) The human SCS heterodimer, with the alpha subunit in orange and the beta subunit in teal (PDB ID: 6G4Q) [22]. All structural images were generated using *UCSF Chimera X*.

Succinyl-CoA synthetase (SCS), or succinate thiokinase or succinyl-CoA ligase, is a mitochondrial enzyme with a primary function in the TCA cycle (Figure 1). The structure and function of SCS have been extensively studied in prokaryotic organisms, particularly in the bacterium *Escherichia coli* (*E. coli*). SCS is well known for catalyzing the reversible conversion of succinyl-CoA to succinate in the fifth step of the TCA cycle, which is also the only catalytic step coupled with substrate-level phosphorylation of either GDP or ADP [11,12]. In *E. coli*, SCS is composed of two subunits, alpha (α) and beta (β), which form a tetrameric quaternary structure consisting of two copies of each subunit [(αβ)2] (Figure 2A) [13,14,15]. The genes encoding the α and β subunits in *E. coli*, *sucD* and *sucC*, respectively, are part of a larger operon driving the transcription of multiple TCA cycle proteins [16]. Research within prokaryotic organisms has been crucial to the understanding of SCS, as structural and functional studies within *E. coli* and other bacteria have provided mechanistic insight into SCS catalysis, including the nucleotide, coenzyme, and succinate binding sites (Figure 2B) [13,17,18,19]. Additionally, the active catalytic sites have been identified at the contact junctions between the α and β subunits (Figure 2C)., where ahistidine residue in the α subunit (His246α) is phosphorylated during the catalytic reaction and stabilized by glutamate residues in both the α (Glu208α) and β (Glu197β) subunits [20,21,22]. This transiently phosphorylated histidine is then hypothesized to swing “down” towards Glu197β where it is involved in the substrate-level phosphorylation at the nucleotide-binding site within the β subunit, [17,18,21]. This catalytic mechanism is also highly conserved across eukaryotes.

### 2.2. SCS in Eukaryotic Organisms: The Presence of Two SCS Isoforms

Although SCS is described as a heterotetramer in Gram-negative bacteria, such as *E. coli*, in Gram-positive bacteria and eukaryotic organisms, SCS has a heterodimeric quaternary structure, with single α and β subunits (Figure 2D) [23,24]. In addition to high sequence homology to prokaryotic SCS, ectopic expression of mammalian SCS subunits rescued enzymatic function in SCS-deficient *E. coli*, suggesting mechanistic conservation of SCS catalysis [25]. Although initially only believed to have specificity for GDP, ADP- and GDP-specific β subunits of SCS, encoded by unique genetic isoforms, have been identified across multiple species [18,26,27,28,29]. In humans, the ADP-specific β subunit is encoded by *SUCLA2* (chromosome 13). The GDP-specific β isoform is encoded by *SUCLG2* (chromosome 3), and both ADP-specific (A-SCS) and GDP-specific (G-SCS) versions of the enzyme, have the same catalytic α subunit, encoded by *SUCLG1* (chromosome 2) [28,29]. Additionally, while SCS is ubiquitously expressed, A-SCS and G-SCS isoforms exhibit differential expression patterns across tissues. Specifically, while G-SCS has higher expression in anabolic tissues such as the liver and kidney, A-SCS is the predominant SCS isoform in tissues with high energy demand, including skeletal muscle and the brain [30]. Furthermore, SUCLA2 is highly expressed as the predominant β-isoform within the brain and neurons [30,31]; therefore, genetic perturbations within A-SCS severely affect neuronal function.

### 2.3. The Biological Significance of SCS: Beyond the TCA Cycle

The TCA cycle was first postulated by Hans Adolf Krebs in 1937 [32], and the specific catalytic role of SCS within this cyclic chain of reactions was studied in the 1950s [26]. Since this discovery, the SCS enzyme and its corresponding metabolic intermediates have been associated with many other metabolic and biological processes within and outside the mitochondrial matrix. Within the TCA cycle, the interconversion of succinyl-CoA and succinate catalyzed by SCS also serves as an entry point for amino acid and fatty acid catabolism (Figure 1). Propionyl-CoA is generated from odd-chain fatty acids and the catabolism of isoleucine, threonine, methionine, and valine. Propionyl-CoA is converted to methylmalonyl-CoA by propionyl-CoA carboxylase, and methylmalonyl-CoA mutase then catalyzes the conversion of methylmalonyl-CoA to succinyl-CoA for entry into the TCA cycle, thus serving as a critical intersection point of glucose, amino acid, and fatty acid metabolism [33,34]. Succinyl-CoA is also involved in processes beyond the TCA cycle. For example, succinyl-CoA is the coenzyme A donor in the breakdown of ketones [35] and is a critical intermediate in heme synthesis [36]. Additionally, SCS-mediated regulation of succinyl-CoA has been associated with changes in lysine succinylation, a protein modification linked to widespread metabolic and epigenetic effects [37,38]. In addition to increased protein succinylation, loss of SCS has also been associated with mtDNA depletion, suggesting that SCS also plays a critical role in mtDNA maintenance [10,39]. Perturbed mtDNA maintenance and protein succinylation are two of the molecular pathways associated with pathogenesis in patients with SCS-deficient encephalomyopathy explored within this review.

## 3. The Genetic and Phenotypic Spectrum of Human SCS Deficiency

### 3.1. Human SCS Variants

There have been clinical reports of 64 patients with pathogenic variants in *SUCLA2* (Table 1) and 33 patients with pathogenic variants in *SUCLG1* (Table 2). At the time of writing this review, there have been no published reports of disease-causing variants in *SUCLG2*. The first cases of SCS-related encephalomyopathy were reported in 2005 in a small consanguineous Middle Eastern pedigree with biallelic variants in *SUCLA2* [10]. The two patients described were compound heterozygous for a complex genetic rearrangement, leading to partial deletion of exon six and the intronic junction between exons 6 and 7, and a 5-nucleotide insertion, also leading to the skipping of the sixth exon. Shortly afterwards, a study of 12 patients from the Faroe Islands, where there is a higher incidence of inherited mitochondrial encephalomyopathy (1:1700), revealed a founding splice site mutation (IVS4+1G>A) leading to skipping of exon 4 [39]. This population has been one of the most highly studied in investigations of SCS-related encephalomyopathy. Since these initial reports, 24 additional variants in *SUCLA2* have been associated with metabolic encephalomyopathy (Table 1), with the majority causing missense mutations within conserved residues of SUCLA2, particularly within the ATP-grasp domain and the CoA-ligase domain, likely disrupting either the stability of the dimer or the catalytic activity through substrate level phosphorylation [40,41]. Similarly, disease-associated variants in *SUCLG1* were first reported in a small consanguineous pedigree of Pakistani origin, describing three patients with homozygous deletions of 2 nucleotides within exon 2 (c.113_114delAT) [42]. Since then, many clinical reports have been published on single patients with *SUCLG1* variants, most leading to changes in conserved amino acids within the protein (Table 2). The reported variants have led to a wide clinical spectrum in SCS deficiency; however, all patients presented with diagnosed metabolic encephalopathy.

### 3.2. The Clinical Characteristics and Diagnosis of SCS-Deficient Encephalomyopathy

Variants in the genetic components of SCS are generally considered pathogenic candidates in patients presenting with mitochondrial disease. Although both primary RC deficiencies and MtDNA Depletion Syndromes have wide genetic heterogeneity and are associated with similar clinical presentation, including progressive, early-onset encephalomyopathy and lactic acidosis, SCS deficiencies are considered likely candidates with the presence of mild methylmalonic aciduria and elevations in C3 and C4-DC acylcarnitines, which support the primary TCA cycle defect [8]. The most common clinical characteristics observed across both SUCLA2- and SUCLG1-deficient patients are severe hypotonia, dystonia, sensorineural hearing impairment, delayed psychomotor development or psychomotor regression, growth deficits, feeding difficulties, and failure to thrive (Table 1 and Table 2). In reports where brain magnetic resonance imaging (MRI) was conducted, many cases reported cerebral atrophy or malformation; however, nearly all patients exhibited hyperintensity lesions in the caudate nuclei and putamen of the basal ganglia (Table 1 and Table 2).The impairment of the basal ganglia, largely involved in motor control and motor learning [68], likely contributes to observed neuropathic motor phenotypes such as delayed psychomotor milestones, dystonic and ataxic movements, and hypotonia. Seizures, ptosis, and ophthalmoplegia are also common findings in SCS-deficient patients (Table 1 and Table 2). Although these signs and symptoms are observed across patients with deficits in both the α and β subunits of SCS, the presentation of SUCLG1 patients is often more severe, with cardiac, hepatic, and renal dysfunction not reported in SUCLA2 patients (Table 2). This likely results from the loss of the ubiquitous α subunit affecting both A-SCS and G-SCS, thus affecting SCS across more tissues. Moreover, Carrozzo et al. performed an informative phenotype-genotype correlation analysis of 71 patients with pathogenic variants in either *SUCLA2* or *SUCLG1*. From their analysis, they reported a median survival of 20 years for SUCLA2 patients, while patients with pathogenic variants in the SCS α subunit only survived an average of two months, suggesting that the multi-organ damage caused by SUCLG1 deficiency led to decreased survivability and poor prognosis [9].

In addition to distinct encephalopathic clinical features, SCS deficiency is commonly diagnosed due to a hallmark metabolic profile. Nearly all clinical cases reviewed report the presence of lactic acidosis, elevations of methylmalonic acid (MMA) in the peripheral blood and/or urine, and many reported elevations of C3 (propionyl-) and C4-DC (succinyl- and methylmalonyl-) carnitines. SCS deficiency has also commonly been described as an MtDNA Depletion Syndrome (MDS), as depletion of the mtDNA copy number has been observed in skeletal muscle and liver biopsies, peripheral blood leukocytes, and patient-derived fibroblasts (Table 1 and Table 2). The depletion of mtDNA is associated with combined RC deficits, most commonly through decreased complex formation and activity of complex I and complex IV, and sometimes complex III (Table 1 and Table 2). The changes in mtDNA content coupled with RC deficiency and clinically significant changes in acylcarnitines are diagnostic flags for SCS deficiency and mark significant points of research opportunities linking these metabolic hallmarks to the clinical presentation of SCS deficiency.

## 4. Research Models of SCS-Related Encephalomyopathy

### 4.1. Cellular Models of SCS-Deficiency

Research investigating pathogenic mechanisms of SCS-related mitochondrial encephalomyopathy has largely relied on in vitro cellular models until recently. More specifically, functional studies have been largely derived from patient-derived fibroblast cells [58,62,69]. For example, Donti et al. observed mtDNA depletion (50% control after multiple cell passages) in a study of fibroblasts derived from a SUCLG1 patient, while muscle biopsy revealed mtDNA elevation. Additionally, transgenic expression of wild-type (WT) *SUCLG1* in the patient fibroblasts completely rescued the observed reduced SCS protein expression, SCS activity, mtDNA content, and cellular respiration defect [62]. In another study of patients with a novel *SUCLG1* variant, studies on patient-derived fibroblasts were performed, validating loss of SCS activity, including substrate-level phosphorylation, and observing RC deficiencies. They also associated the loss of SUCLG1 with perturbed mitochondrial morphology and mis-localization of the GDP-specific β isoform [58]. Even more recently, fibroblast and myotubes derived from patients with SUCLA2 deficiency were used to demonstrate accumulated succinyl-CoA leading to a widespread increase in protein succinylation within the cells [69].

Although these studies have largely been phenotypically descriptive, mechanistic insights have been gleaned from these in vitro investigations. For example, in the 2020 paper published by Gut et al. described above, mass spectrometry was used to identify targets of protein hyper-succinylation, including proteins within major metabolic pathways such as the TCA cycle, OXPHOS, and glycolysis. These data suggest that metabolic perturbations may be at least partially described by increased protein modification, and this dataset will be invaluable in future investigations of succinylation-related pathogenesis [69]. Another study that used RNA interference (RNAi) to knock down *Sucla2* in cultured mouse neurons described A-SCS loss with mtDNA instability and perturbed mitochondrial dynamics, suggesting a role of A-SCS in maintaining mitochondrial integrity within neurons [70]. Expanding on the observations from cellular in vitro studies, the development of in vivo models has also proven to be a crucial resource in SCS-related research.

### 4.2. Animal Models of SCS-Deficiency

Donti, et al. published the first murine model of SUCLA2 deficiency in 2014 following the identification a *Sucla2* gene trap allele in a screen for abnormal mitochondrial phenotypes in mouse embryonic stem cells [71]. Constitutive homozygous mutations in *Sucla2*, however, resulted in late-gestational embryonic lethality. Data collected in embryonic tissue and mouse embryonic fibroblasts (MEFs), nonetheless, modeled SCS-deficient phenotypes seen in patients, including elevated MMA and progressive mtDNA depletion within the brain and skeletal muscle, leading to RC deficiencies in complexes incorporating mtDNA-encoded proteins. While the modeling of human molecular phenotypes demonstrates the utility of an in vivo model, assessing whole animal phenotype and observable evidence of encephalomyopathy proves difficult in the context of embryonic lethality. Kacso et al. generated viable double heterozygous mouse models with monoallelic variants in both *Sucla2* and *Suclg2*, and while this model provides useful information on the dynamics of the SCS β subunits in different tissues in the heterozygous state, it does not result in the loss of A-SCS or G-SCS that could lead to severe phenotypes associated with SCS-deficient mitochondrial disease in humans [72].

In their paper identifying succinylation targets in SUCLA2-deficient patient fibroblasts, Gut et al. also developed a zebrafish model of Sucla2 deficiency [69]. While the zebrafish model also exhibited embryonic lethality, surviving larvae were studied to correlate changes in protein succinylation with mitochondrial pathogenesis, which will be reviewed in detail in this review’s next section. Recently, a conditional knockout of *Sucla2* in the mouse forebrain using the Cre-Lox system was reported [73]. This model allows for the study of brain-specific SCS-deficient pathogenesis in vivo in adult mice. As reported in this study, SCS deficiency resulted in global hyper-succinylation linked to observed neuronal and metabolic phenotypes corroborating the importance of considering protein succinylation as a prominent feature and potential pathomechanism of metabolic encephalopathy.

## 5. Pathogenic Mechanisms Requiring Further Exploration

### 5.1. Mechanisms of SCS-Related MtDNA Maintenance

In publications describing patients with SCS deficiency, depletion of mtDNA in skeletal muscle and patient-derived fibroblasts is a commonly observed phenotype (Table 1 and Table 2) [74,75]. However, the relationship between this TCA-cycle enzyme and pathways of mtDNA maintenance is not fully understood. The prevailing hypothesis in the field postulates that SCS deficiency disrupts the maintenance of mitochondrial nucleoside triphosphate (dNTP) pools serving as the building blocks of mtDNA [10,74]. In contrast to nuclear DNA, the mitochondrial chromosome has a higher turnover rate and replicates throughout all stages of the cell cycle [76]. Because dNTP synthesis does not occur *de novo* within mitochondria, mtDNA maintenance depends upon the regulation of the nucleotide salvage pathway [10,76]. Mitochondrial NDPK (mtNDPK), encoded by nuclear gene *NME4*, catalyzes the reversible phosphorylation of nucleoside diphosphates (dNDPs) to dNTPs within mitochondria and is, therefore, a crucial enzyme in the regulation of internal mitochondrial dNTP pools [77]. Interestingly, SCS has been shown to bind directly to mtNDPK [74,78]. Furthermore, significantly reduced expression of mtNDPK was observed upon *Sucla2* knock-down in mouse neurons [70].

However, whether and how the interaction between SCS and NDPK is disrupted in SCS-related mtDNA depletion is still unknown. One possibility is that the variants in SCS produce a conformational change preventing its association with mtNDPK. Alternatively, many missense variants where mtDNA depletion was observed are predicted to either destabilize the interaction between the α and β subunits of SCS or lead to protein misfolding, while variants in patients without observed mtDNA depletion were not predicted to alter complex formation [40,41]. It’s possible, therefore, that mtNDPK activity is dependent upon forming a stable complex with SCS or that the loss of interaction with SCS destabilizes mtNDPK expression or activity [70]. Another hypothesis is that canonical SCS activity is required for mtDNA maintenance, as many missense variants in *SUCLA2* and *SUCLG1* resulting in mtDNA depletion are localized to the ADP-binding domain or CoA-ligase domain and are predicted to have deleterious effects on SCS catalytic activity [40,41,55]. NDPK-mediated phosphorylation of dNDPs occurs through a catalytic phosphohistidine intermediate [76], similarly to SCS-mediated substrate-level phosphorylation. It is, therefore, possible that SCS donates a phosphate or dNDP to mtNDPK via its catalytic mechanism within the TCA cycle, and disrupting this mechanism results in the loss of mtNDPK activity. On the other hand, *Sucla2* knock-down in cultured neurons was also accompanied by reduced expression levels of mitochondrial DNA polymerase γ and the mtDNA helicase, Twinkle, proteins essential for mtDNA replication, suggesting yet another potential mechanism to explore [70].

While SCS-related mtDNA maintenance is a crucial avenue to investigate, mtDNA depletion is not always present within SCS patients, and there is a similar variability in mtDNA content observed in the available research models [58,62,69,70,71,72,73]. More specifically, mtDNA depletion has only been reported in skeletal muscle, liver, peripheral blood leukocytes, or patient-derived fibroblasts (Table 1 and Table 2). MtDNA content has not yet been described from the brain of SCS-deficient patients. The data from research models are also variable regarding mtDNA content in the brain in the context of SCS deficiency. While mtDNA depletion was observed in *Sucla2* knock-down in cultured neurons [70] and brain during late gestation in constitutive mouse *Sucla2* mutants [71], there was no mtDNA depletion observed in the cortex of the forebrain-specific mouse knock-out; instead, a modest elevation of mtDNA content was observed in the adult mutant cortex [73]. The lack of mtDNA depletion observed within the conditional knockout model may result from postnatal Cre-recombination-mediated deletion of the *Sucla2* allele; however, this hypothesis requires the assumption that SCS is not required for mtDNA maintenance in post-mitotic neurons. Given the heterogeneity of this phenotype, mtDNA content may not be a consistent hallmark of SCS-deficient encephalopathy; rather, it may present in a tissue-specific manner, as observed by Donti et al. [62], and be influenced by the specific variant(s) inherited and/or yet undetermined genetic or environmental modifiers. Postmortem analysis of brain mtDNA content in patients could be conducted to determine the importance of this pathway within the brain. Furthermore, inconsistent observation of mtDNA depletion within patients and research models underlies the need to consider additional pathomechanisms of SCS-deficient encephalopathy.

### 5.2. Protein Acylation as a Novel Mechanism in SCS-Deficiency

Protein acylation is a post-translational modification of proteins emerging as an essential metabolic regulator. Beyond well-studied acetylation, many other types of protein acylation have been recently described, including malonylation, propionylation, and succinylation [79]. In protein acylation, fatty acyl-coenzyme A (acyl-CoA) species, regulated through the metabolic generation of acyl-CoA intermediates within mitochondria, donate acyl groups to lysine residues, having a prominent effect on the size and charge of the amino acid side chain [79]. Within the cell, protein acylation is enzymatically regulated by the addition of the modifications via lysine acyltransferases (KATs) and their removal by lysine deacylases (KDACs) [80]. However, although mitochondrial pathways are primarily affected by protein acylation, there has been no identified KAT with mitochondrial localization. Therefore, mitochondrial acylation is widely believed to occur nonenzymatically, and much of the current research on mitochondrial protein succinylation is conducted in models of the Sirtuin family of mitochondrial KDACs [80,81].

With the hypothesis of nonenzymatic acylation within the mitochondria, the idea that intramitochondrial accumulation of succinyl-CoA resulting from loss of SCS may impact protein succinylation has started to be investigated. There have been multiple reports of SCS patients lacking mtDNA depletion while exhibiting encephalomyopathy with developmental delay, hypotonia, and even RC deficiencies (Table 1 and Table 2) [51,65]. Of note, these patients also show marked increases in succinyl-, methylmalonyl-, and propionyl-carnitines, which arise from the union of their respective acyl-CoAs with carnitine and are involved in regulating the intramitochondrial concentrations of acyl-CoAs to free CoA [34]. Recent investigations, therefore, have begun to assess protein succinylation within SCS deficiency. After identifying nearly 1000 protein succinylation targets within SUCLA2-deficient patient-derived fibroblasts, Gut et al. investigated the functional significance of hyper-succinylation in *sucla2*-knockout zebrafish larvae. In these larvae, over-expression of SIRT5, the mitochondrial desuccinylase enzyme, resulted in partial rescue of reduced mitochondrial respiration without rescuing observed mtDNA depletion [69]. Similarly, hypersuccinylation of NADH dehydrogenase (Complex I in the electron transport chain) was associated with functionally reduced activity within the cerebral cortex of the forebrain-specific *Sucla2* knock-out mice without depletion [73]. These results suggest that protein succinylation is at least partially responsible for the metabolic perturbations of SCS-deficient respiratory deficiencies.

In addition to hypothesized pathogenesis in SCS deficiency, altered protein succinylation has recently been associated with other metabolic and neuropathic diseases. Altered protein succinylation patterns have been observed after brain injury in intracerebral and subarachnoid hemorrhage and stroke [82,83,84] and within the brains of Alzheimer’s patients [85]. Changes in protein succinylation have also been linked to the pathogenesis of metabolic disorders such as diabetes [86,87] and certain cancers [88,89,90]. Furthermore, a growing body of literature provides evidence for the presence of anaplerotic enzymes, such as SCS, outside of the mitochondria, linking acyl-CoA metabolism to histone acylation and epigenetic changes in chromatin regulation and gene expression [37,73,91,92,93,94]. These findings suggest that protein succinylation within and outside the mitochondria plays a crucial regulatory role in metabolism and neurodegenerative disease. These observations underscore the importance of studying the downstream consequences of perturbed protein succinylation as a pathomechanism in SCS-deficient encephalopathy and other more common neurological disorders.

## 6. Conclusions and Perspectives

The understanding of SCS deficiency as an etiology of mitochondrial encephalomyopathy has significantly advanced since the first patients were reported in the early 2000s. While the connection between primary TCA cycle dysfunction and pathological mechanisms of mitochondrial encephalomyopathy was poorly understood, patient reports and recently developed research models have provided invaluable insights into the mechanisms linking SCS-deficient genotypes to phenotypes. An updated compilation of the symptoms associated with variants in both the α and β subunits of SCS provides a broader understanding of the relationship between SCS structure, function, and phenotype. Moreover, newly available animal models are a crucial resource as researchers begin to link molecular mechanisms of SCS deficiency to whole animal phenotypes by allowing the opportunity to study animal behavior and physiology in vivo.

Although mtDNA depletion and protein succinylation have been identified as promising molecular mechanisms warranting further investigation, much of the research up to this point has necessarily been descriptive and is generating many questions to be answered. For example, although the NDPK-associated hypothesis for SCS-related mtDNA maintenance has been established for many years, additional studies will be required to confirm this theory, and other potential contributing mechanisms should be investigated. Additionally, as the identification of protein succinylation as a new pathogenic target in SCS deficiency is relatively recent, this area of research is in its early stages. For example, it is still not yet fully understood how negatively charged succinyl-CoA crosses the mitochondrial membranes to modify extramitochondrial proteins [94].

Furthermore, while researchers have begun to study the consequences of lysine succinylation in SCS deficiency with the help of large-omic datasets, the effects of other protein acylations, such as methylmalonylation and propionylation, remain unexplored. These recent developments also raise the possibility of modulating KDACs as a novel therapeutic strategy for SCS deficiency. With the potential for many avenues of research related to SCS deficiency, researchers are well-positioned to improve global understanding of the relationship between perturbed metabolic pathways and mitochondrial and neuronal dysfunction, putting the field in a promising position for the development of therapeutic options for mitochondrial encephalomyopathies.

## Figures and Tables

**Table 1:** Published clinical variants in *SUCLA2* and associated phenotypes.

**Genotype**	Genomic Rearrangement with deletion of 43nt + c.789_802+29delinsATAAA	Homozygous IVS4+1G>A	Homozygous c.850C>T	c.850C>T + c.352G>A		Homozygous c.534+1G>A *	Homozygous c.308C>A	c.1048G>A + c.1049G>T	Homozygous c.751G>A	Homozygous c.998A>G	c.998A<G + 1.54Mb Deletion in 13q14	Homozygous c.970G>A	Homozygous c.985A>G
**Gene Effect**	Skip Exon 6, Frameshift	Skip Exon 4 (null)	p.Arg284Cys	p.Arg284Cys + p.Gly118Arg		Multiple Exon Skipping (null)	p.Ala103Asp	p.Gly350Ser + p.Gly350Val	p.Asp251Asn	p.Asp333Gly	p.Asp333Gly + 7 Gene Deletion	p.Gly324Ser	p.Met329Val
**N**	4	12	2	1		12	2	2	2	5	1	1	1
**Clinical Characteristics**													
**Cerebral Atrophy**	+ (1/2)	+ (7/8)	+ (2/2)	+		+ (8/8)	+	+ (2/2)		+ (3/4)	+	+	+
**Basal Ganglia Lesions**	+ (2/2)	+ (4/8)	+ (2/2)	+		+ (7/7)	+ (2/2)		+ (2/2)	+ (2/4)	+	+	
**Delayed/Demyelination**		+ (1/8)				+ (2/11)							-
**Ataxia**								+ (2/2)					
**Chorea**										+ (2/5)	+		
**Sensorineural Hearing Impairment**	+ (2/2)	+ (10/12)	+ (2/2)			+ (12/12)	+ (2/2)	+ (2/2)	+ (2/2)	+ (2/5)	+	+	+
**Seizures**	+ (2/2)	+ (1/12)				+ (1/111)			-	-	+		
**Dystonia**	+ (1/2)	+ (12/12)	+ (2/2)	+		+ (10/11)	+ (2/2)	+ (1/2)	+ (2/2)	+ (3/5)	+		+
**Hyoptonia**	+ (2/2)	+ (12/12)	+ (2/2)	+		+ (12/12)	+ (2/2)	+ (2/2)	+	+ (5/5)	+	+	+
**Muscular Atrophy**		+ (12/12)					- (2/2)	+ (1/2)		+ (1/5)	+	+	+
**Histochemical Myopathy**	- (2/2)	+ (7/7)								+ (2/5)	+		
**Delayed Psychomotor Development**	+ (2/2)	+ (12/12)	+ (2/2)	+		+ (11/11)	+ (2/2)	+ (2/2)	+	+ (5/5)	+	+	+
**Growth Deficit**		+		+			+ (2/2)	+ (1/2)		+ (4/5)	-	+	+
**Feeding Difficulties**		+	+ (2/2)	+		+ (11/12)	+ (2/2)	+ (1/2)	+ (2/2)	+ (4/5)		+	+
**Vomiting**							+ (2/2)						+
**Headaches**													
**Failure to Thrive**			+ (1/2)	+		+ (10/11)	+	+ (2/2)	+	+ (4/5)		+	+
**Ptosis**	+ (1/2)	+ (2/12)	-	-		+ (10/11)	+ (2/2)		-	+ (1/5)	+		
**Gastroesophageal Reflux**	+ (1/2)												
**Gastrointetinal Issues**			+ (2/2)	+		+ (10/11)	+						+
**Respiratory Distress**	+ (2/2)	+					+			+ (4/5)		+	
**Cardiomyopathy**						+ (1/11)			+				-
**Hepatopathy**													-
**Renal Symptoms**						+ (1/11)			+				-
**Anemia**	+ (2/2)												
**Metabolic Profile**													
**Lactic Acidosis**	+ (2/2)	+ (7/8)	+ (2/2)	+		+ (7/9)	+ (2/2)	+ (2/2)	+ (1/1)	+ (2/5)	+	+	+
**Mild Methylmalonic Aciduria**		+ (10/10)	+ (1/1)	+		+ (4/5)	- (1/1)	+ (2/2)	- (1/1)	Mild to Normal	+		+
**Elevated C3 Acylcarnitine**			+ (1/1)	+		+ (3/3)		+ (1/1)				+	
**Elevated C4DC Acylcarnitine**			+ (2/2)	+		+ (3/3)		+ (1/1)	+ (1/1)			+	+
**MtDNA Content**	Depleted in Muscle Normal in Fibroblasts	Depleted in Muscle (3/3)	Depletion in Muscle (1/1) Depletion in Fibroblasts (1/1)	Depletion in Muscle Depletion in Fibroblasts		Normal in Fibroblasts (1/1)	Depletion in Muscle (2/2)	Depleted in Muscle (1/1)		Normal in Muscle (1/2) Mild Depletion in Muscle (1/2)		+	Moderate Depletion in Fibroblasts Not Measured in Muscle
**Respiratory Chain Defects**	+ (2/2)	+					+ (2/2)	+ (1/1)		+ (2/5)	+		
**Reference**	[10]	[39]	[43]	[43]		[9,43]	[44]	[9,45]	[40]	[9,41]	[41]	[46]	[47]

**Genotype**	Homozygous c.1219C>T	Homozygous 258kb Deletion	c.1106dupA + 46kb deletion	Homozygous c.920C>T	Homozygous c.750C>A	c.160_161insAGA + c.850C>T	c.1204delA + c.308C>T	Homozygous c.1271delG	c.534+1G>A + c.985A>G	Homozygous c.83delC	Homozygous c.1276C>T	c.985A>G + c.920C>T	Homozygous Mutations c.851G>A; c.971G>A
**Gene Effect**	p.Arg407Trp	Whole Gene Deletion	p.Val370Glyfs*18 + Deletion of Exons 1-5 (null)	p.Ala307Val	p.Tyr250*	p.Ser54* + p.Arg284Cys	p.Ile402Tyrfs*18 + p.Ala103Asp	p.Gly424Aspfs*18	Multiple Exon Skipping (null) + p.Met329Val	p.Ala28Valfs*32	p.Arg407Trp	p.Met329Val + p.Ala307Val	p.Arg284His; p.Gly324Asp
**N**	6	1	2	1	1	1	1	1	3 *	1	1	2	2
**Clinical Characteristics**													
**Cerebral Atrophy**	+ (2/3)	+	+ (2/2)	+	+	+	+	+		+		+ (1/1)	
**Basal Ganglia Lesions**	+ (4/4)	+	+ (2/2)	+		+	+	+	+ (2/2)	+		+ (1/1)	
**Delayed/Demyelination**													
**Ataxia**										+	+		
**Chorea**	+ (6/6)	+									+	+ (2/2)	
**Sensorineural Hearing Impairment**	+ (6/6)		+ (2/2)	+	+	+	+	+	+ (3/3)	+	+	+ (2/2)	+
**Seizures**	+ (1/3)		+ (1/2)	+									+
**Dystonia**	+ (6/6)	+		+		+	+	+	+ (1/2)	+		+ (2/2)	+
**Hyoptonia**	+ (3/3)	+	+ (2/2)	+	+	+	+	+	+ (2/3)	+	+	+ (2/2)	+
**Muscular Atrophy**		+	+ (2/2)			+			+ (1/2)				
**Histochemical Myopathy**	- (4/4)		+ (2/2)		+	+					+	+ (2/2)	
**Delayed Psychomotor Development**	+ (3/3)	+	+ (2/2)	+	+	+	+	+	+ (3/3)	+	+	+ (2/2)	+
**Growth Deficit**			+ (2/2)	+	+							+ (2/2)	+
**Feeding Difficulties**	+ (1/3)	+	+ (2/2)	+	+	+	+	+	+ (2/3)			+ (2/2)	+
**Vomiting**	+ (1/3)		+ (1/2)	+				+			+	+ (1/2)	
**Headaches**											+	+ (1/2)	
**Failure to Thrive**	+ (1/3)	+			+		+	+	+ (1/2)	+			+
**Ptosis**	+ (1/3)				+			+	+ (1/2)			+ (1/2)	+
**Gastroesophageal Reflux**	+ (1/3)		+ (1/2)	+	+							+ (1/2)	
**Gastrointetinal Issues**								+			+	+ (2/2)	
**Respiratory Distress**				+		+	+		+ (1/3)			+ (1/2)	
**Cardiomyopathy**													
**Hepatopathy**													+
**Renal Symptoms**													
**Anemia**												+ (1/2)	
**Metabolic Profile**													
**Lactic Acidosis**	+ (6/6)	+	+ (2/2)	+	+	-	+	+	+ (3/3)	+	+	+ (2/2)	+
**Mild Methylmalonic Aciduria**	+ (5/6)	+	+ (2/2)	+	+	+	+	+	+ (3/3)	+		+ (2/2)	
**Elevated C3 Acylcarnitine**	+ (3/3)											+ (2/2)	
**Elevated C4DC Acylcarnitine**	+ (3/3)											+ (2/2)	
**MtDNA Content**	Depletion in Muscle (1/1) Normal in Muscle (3/3)							Mild Depletion in Muscle			Depletion in Muscle	Normal to Elevated in Muscle	
**Respiratory Chain Defects**	- (4/4)	+	+ (1/1)	+	+	+		+	-		+	+ (2/2)	
**Reference**	[9,48]	[9]	[9]	[9]	[9]	[9]	[9]	[9]	[9]	[49]	[50]	[51]	[52]

All known and reviewed published literature describing patients with pathogenic variants in *SUCLA2* are provided. Columns are organized by reported *SUCLA2* genotype, with novel variants described within the clinical reports outlined in the first section of the table. Clinical characteristics described within each report are denoted by “+”, and “-” indicates that the authors explicitly mention the absence of the relevant phenotype. Where multiple patients with the same genotype are described, the number of patients exhibiting the associated phenotype among the total number of patients in which the phenotype was measured is also highlighted. References are included at the bottom of the table, and interactive excel tables have been provided in Supplemental File “VariantTables”. * Note: One patient was heterozygous for c.534+1G>A; however, the second allele was not identified.

**Table 2:** Published clinical variants in *SUCLG1* and associated phenotypes.

**Genotype**	Homozygous c.113_114delAT	Homozygous c.215G>C (Also reported as c.254G>C)	Homozygous c.40A>T	c.509C>G + c.97+3G>C	c.448C>T + Unidentified Variant	Homozygous c.626C>A	c.309_310delTG + c.428T>G	Homozygous c.137C>T		c.626C>A *	Homozygous c.280-1G>A	c.41T>C + c599C>T	Homozygous c.749A>G	
**Gene Effect**	Deletion in Exon 2	p.Gly72Ala (Also reported as p.Gly85Ala)	p.Met14Leu	p.Pro170Arg + Skip Exon 1	p.Gln150*	p.Ala209Glu	p.Thr103fs + p.Ile143Ser	p.Ser46Phe		p.Ala209Glu	Splice Variant	p.Met14Thr + p.Ser200Phe	p.Glu263Gly	
**N**	3	3	1	1	1	4	1	2		1	1	1	1	
**Clinical Characteristics**														
**Cerebral Atrophy**	+ (3/3)	+ (2/3)	+		+	+ (2/2)	+			+	+	+	+	
**Basal Ganglia Lesions**		+ (3/3)	+	+	+	+ (2/2)	+	+ (2/2)		+	+			
**Delayed/Demyelination**		+ (1/3)												
**Ataxia**		+ (1/3)				+ (1/4)				+				
**Chorea**														
**Sensorineural Hearing Impairment**		+ (2/3)	+			+ (1/2)	-	+ (1/2)		+		+		
**Seizures**						+ (1/4)								
**Dystonia**		+ (1/3)	+			+ (2/4)	+	+ (2/2)		+				
**Hyoptonia**	+ (2/3)	+ (3/3)	+	+	+	+ (4/4)	+	+ (2/2)		+		+		
**Muscular Atrophy**		+ (2/3)	+		+	+ (1/1)		+ (2/2)						
**Histochemical Myopathy**	- (1/1)			+	+		+	+ (1/1)			+	-	+	
**Delayed Psychomotor Development**		+ (3/3)		+	+	+ (2/4)	+	+ (2/2)		+				
**Growth Deficit**	+ (3/3)	+ (2/3)				+ (2/4)	+			+	+			
**Feeding Difficulties**		+ (2/3)	+		+	+ (1/3)	+	+ (2/2)				+		
**Vomiting**		+ (1/3)	+											
**Headaches**														
**Failure to Thrive**		+ (2/3)			+									
**Ptosis**						+ (1/3)		- (2/2)						
**Gastroesophageal Reflux**		+ (1/3)						+ (1/2)						
**Gastrointetinal Issues**								+ (1/2)						
**Respiratory Distress**	+ (3/3)		+	+	+	+ (4/4)		+ (2/2)		+	+	+		
**Cardiomyopathy**						+ (3/4)		- (2/2)		+	+	+	+	
**Hepatopathy**	+ (1/3)		+			+ (3/3)	+	+ (1/2)				+	+	
**Renal Symptoms**						+ (2/3)		- (1/2)				+	+	
**Anemia**														
**Metabolic Profile**														
**Lactic Acidosis**	+ (3/3)	+ (2/3)	+	+	+	+ (4/4)	+	+ (2/2)		+	+	+	+	
**Mild Methylmalonic Aciduria**		+ (2/3)			+	+ (3/3)	+	+ (2/2)			+	+	+	
**Elevated C3 Acylcarnitine**			+	+		+ (2/3)	+	+ (1/1)			+	+	+	
**Elevated C4DC Acylcarnitine**			+	+		+ (3/3)	+	+ (1/1)			+	+		
**MtDNA Content**	Depletion in Liver (1/3) Depletion in Muscle (1/3)	Mild Depletion in Muscle (1/1)	Depletion in Liver	Depletion in Muscle	Depletion in Muscle	Depletion in Muscle (4/4) Depletion in Liver (3/3)	Depletion in Muscle	Elevated in Muscle		+	Depleted in Muscle	Normal in Muscle Normal in Fibroblasts	Depleted in Muscle	
**Respiratory Chain Defects**	+	+ (2/3)		+	+	+	+	+ (1/2)			+	+	+	
**Reference**	[42]	[9,53]	[54]	[55]	[55]	[56,57,58]	[56]	[9,56]		[58]	[59]	[60]	[61]	

**Genotype**	Homozygous c.212A>G	c.787G>A + c.626C>A	c.40A>T + c.635A>G	c.826-2A>G + c.809A>C	c.826-2A>G + c.550G>A	c.961C>G + c.751C>T	Homozygous c.41T>C		Homozygous c.512A>G	c.40A>G + Deletion	Homozygous Mutations c.916G>T; c.619T>C; c.980dupT			c.601A>G + c.871G>C
**Gene Effect**	p.His71Arg	p.Glu263Lys + p.Ala209Glu	p.Met14Leu + Gln212Arg	Splice Variant + p.Lys207Trp	Splice Variant + p.Gly184Ser	p.Ala32Pro + p.Gly251Ser	p.Met14Thr		p.Asn171Ser	p.Met14Val + Deletion Exons 6-9	p.Gly306Ter; p.Tyr207His; p.Met327Ilefs*15			p.Arg201Gly + p.Ala291Pro
**N**	1	1	1	1	1	1	2		1	1	3			1
**Clinical Characteristics**														
**Cerebral Atrophy**			-		+		+ (1/1)			+				+
**Basal Ganglia Lesions**		+	-		+	+	+ (1/1)		+					
**Delayed/Demyelination**														
**Ataxia**			+						+					+
**Chorea**	+		+	+	+	+								
**Sensorineural Hearing Impairment**	+		+				+ (2/2)				+			
**Seizures**					+		- (2/2)				+			
**Dystonia**	+			+	+	+	- (2/2)				+			+
**Hyoptonia**		+	+	+	+	+	+ (2/2)		+		+			+
**Muscular Atrophy**		+					+ (2/2)							
**Histochemical Myopathy**	+		+											
**Delayed Psychomotor Development**		+	+	+	+	+	+ (2/2)				+			+
**Growth Deficit**					+		+ (2/2)		+		+			
**Feeding Difficulties**	+	+		+	+	+	+ (2/2)				+			
**Vomiting**		+					- (2/2)		+	+				+
**Headaches**														
**Failure to Thrive**		+		+	+	+	+ (2/2)				+			
**Ptosis**	-						+ (2/2)				+			
**Gastroesophageal Reflux**		+												
**Gastrointetinal Issues**														+
**Respiratory Distress**		+					+ (2/2)			+				+
**Cardiomyopathy**	-		-				- (2/2)			+				+
**Hepatopathy**							+ (2/2)				+			
**Renal Symptoms**							- (2/2)							+
**Anemia**														
**Metabolic Profile**														
**Lactic Acidosis**	+	+	-	+	+	+	+ (2/2)		+	+	+			+
**Mild Methylmalonic Aciduria**	+	+	+	+	+	+	+ (2/2)		+	+				+
**Elevated C3 Acylcarnitine**				+	+	+	+ (1/1)		+	+				
**Elevated C4DC Acylcarnitine**				+	+	+	+ (1/1)		+	+				
**MtDNA Content**			Normal in Muscle Elevated to Depleted in Fibroblasts	Normal in Leukocytes	Normal in Leukocytes	Normal in Leukocytes	Depletion in Leukocytes		Normal in Muscle					Depleted in Leukocytes
**Respiratory Chain Defects**	-	+	+		+	+			+					
**Reference**	[9]	[9]	[62]	[63]	[63]	[63]	[64]		[65]	[66]	[52]			[67]

All known and reviewed published literature describing patients with pathogenic variants in SUCLG1 are outlined. Novel variants at the time of publication of the clinical reports are provided in the first section of the table, with columns organized by individual SUCLG1 genotype. Clinical characteristics described within each report are denoted by “+”, and “-” indicates that the authors explicitly mention the absence of the relevant phenotype. Where multiple patients with the same genotype are described, the number of patients exhibiting the associated phenotype out of the total number patients in which the phenotype was measured is also highlighted. References are included at the bottom of the table, and interactive excel tables have been provided in Supplemental File. * Note: One patient was described with a heterozygous genomic variant (c.626C>A) with monoallelic expression after cDNA sequencing.

## Data Availability

No new data were created or analyzed in this review. Data sharing is not applicable to this article.

## Supplementary Materials

The following supporting information can be downloaded at: https://www.mdpi.com/article/10.3390/ijms241310725/s1. Supplemental Resources are provided for the clinical review tables.
